# Supplementary figures and images for: Mechanisms of HIV-1 evasion to the antiviral activity of chemokine CXCL12 indicate potential links with pathogenesis
Source: PLoS Pathog. 2021 Apr 19;17(4):e1009526. doi: 10.1371/journal.ppat.1009526 (PMC8084328; doi:10.1371/journal.ppat.1009526)

## Slide 1
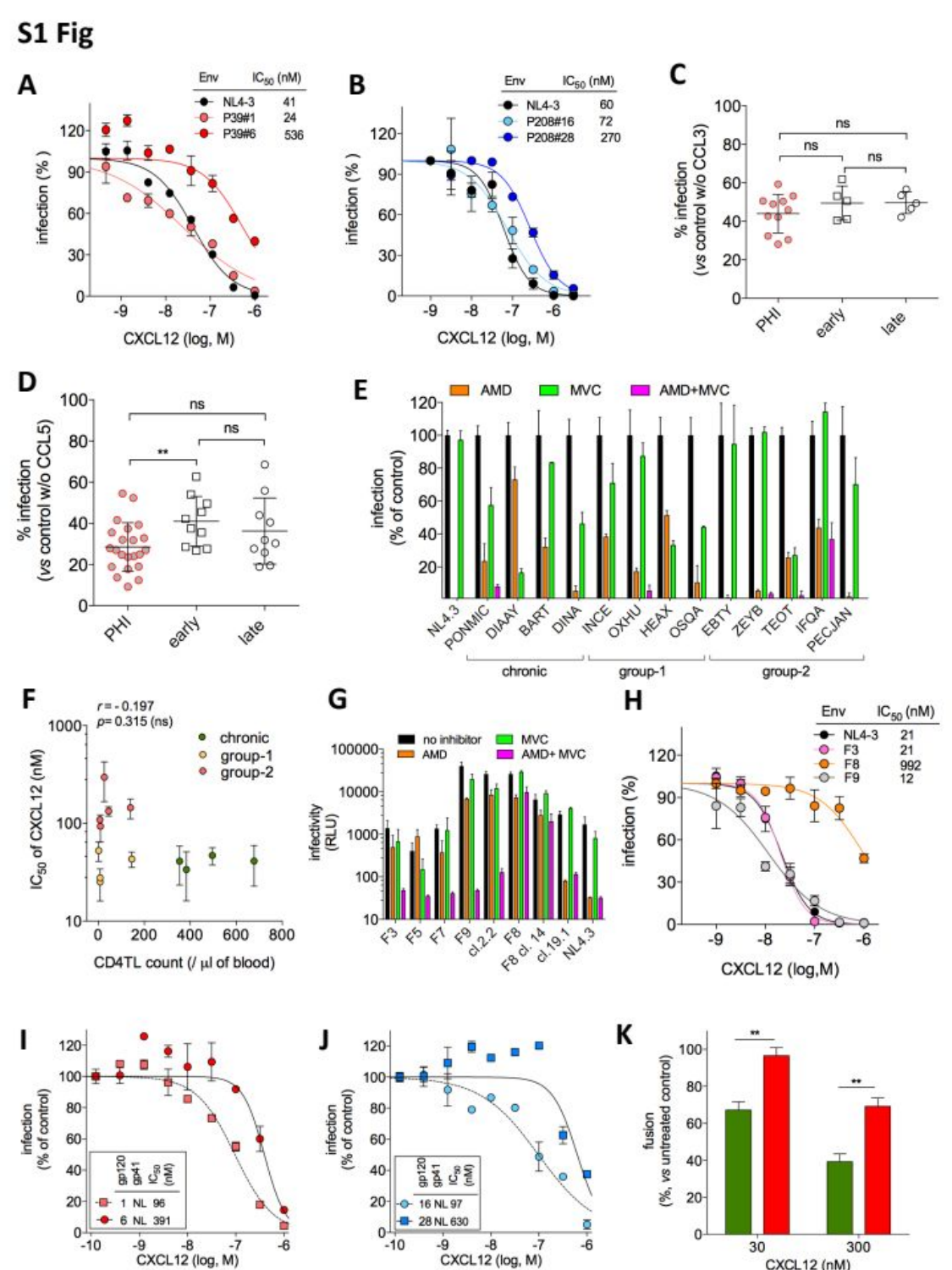

Supplement: S1 Fig — A and B Inhibition by CXCL12 of early (viruses # 1 and 16) and late (viruses # 6 and 28) Envs from PBMCs of two patients of the ACS (P#39 (A) and P#208 (B)) (Related to Fig 1A). Data points (means ± SEM of triplicate determinations) are expressed as percent infection of CD4TL relative to control infection measured in the absence of CXCL12 (100%) and were fitted to a sigmoidal dose-response model with a variable slope. IC50s of CXCL12 were derived from inhibition curves using GraphPad Prism 6. Representative experiments out of at least three independent experiments carried out on CD4TL from distinct healthy donors are shown. C and D Percent infection of PHA/IL-2-activated PBMCs from healthy donors with 20 ng p24 of viruses pseudotyped with Envs isolated at the stage of PHI or the early or late R5 Envs shown in Fig 1B, in the presence of 20 nM CCL3 (C) or 10 nM CCL5 (D). Each data point represents the mean infection measured for a given virus (n = 3 determinations), expressed relative to infection in the absence of chemokine (100%). One (C) and two (D) out of three independent experiments are shown. Error bars represent the SD to the means. ns, not significant; **P < .01 in the Mann-Whitney test. E Coreceptor usage of recombinant virus populations pseudotyped with the Envs depicted in Fig 1C, isolated at the time of diagnosis, at the chronic or late stage of infection. Results (means ± SEM of triplicate determinations) represent infectivity of viruses measured 48 h post-inoculation of R5X4JT cells (with 20 ng of p24), in the presence or absence (black bars, 100%) of 10 μM maraviroc (MVC, light green bars), 10 μM AMD3100 (AMD, orange bars) or a mixture of both antagonists at 10 μM each (pink bars). A representative experiment out of three is shown. Group-1 and group-2 refer to late Envs that are not, or are, significantly more resistant to inhibition by CXCL12, as compared to chronic Envs and NL4-3. F Correlation analysis (Spearman two-tailed test) between the IC50s of [file ppat.1009526.s001.pptx]

## Slide 1
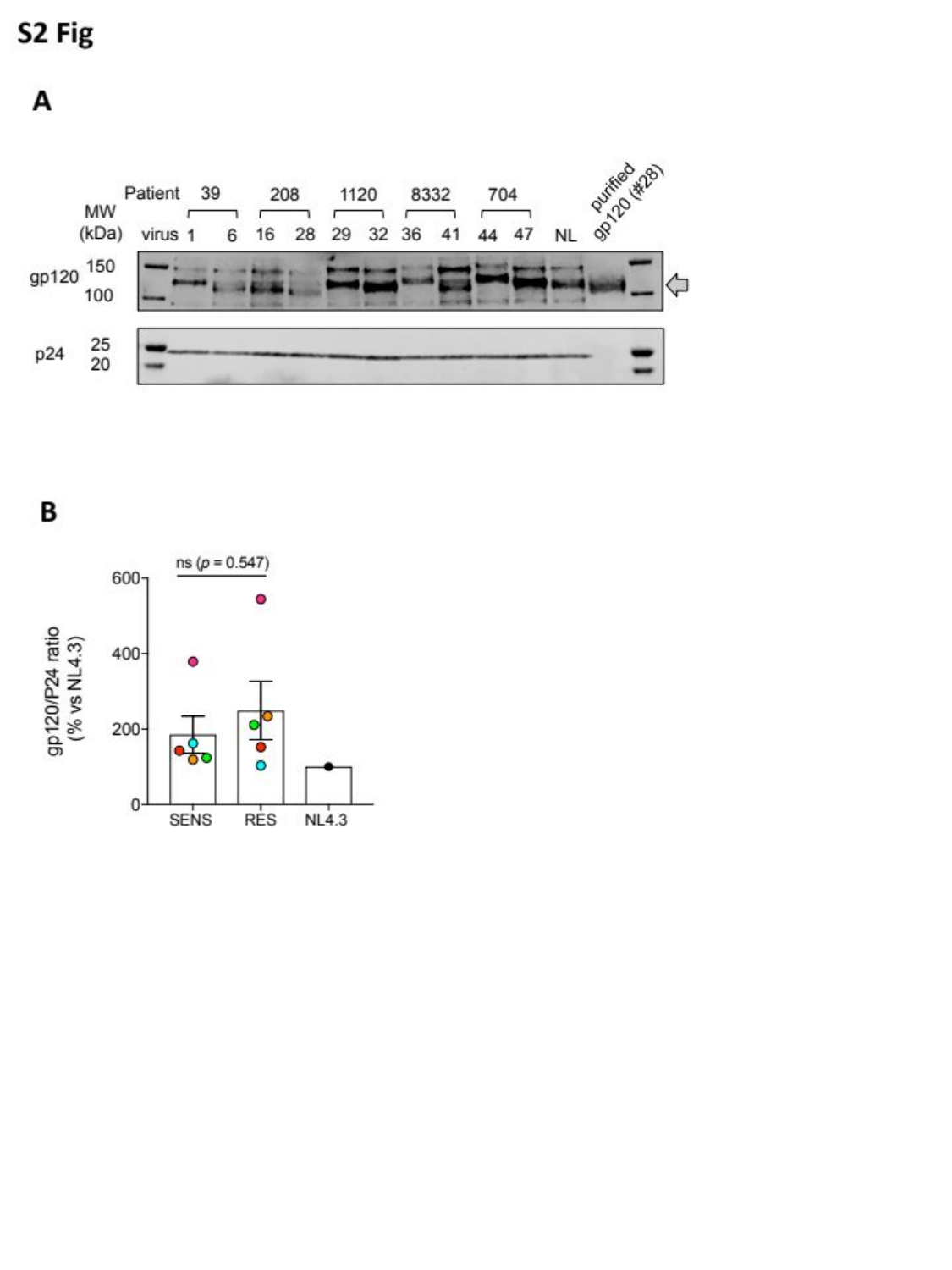

Supplement: S2 Fig — A Western blot analysis of gp120 and p24 expression into viruses pseudotyped with early (SENS) Envs (#1, 16, 29, 36 and 44), late (RES) Envs (#6, 28, 32, 41 and 47) or NL4-3 Env. Three hundred ng of Gag p24 of the different viruses were solubilized in lysis buffer containing XT sample buffer (Biorad), Invitrogen NuPAGE sample reducing agent and 1% Triton X-100, incubated for 5 min at 70°C and then loaded onto Biorad Criterion XT 4–12% Bis-Tris gels under reducing conditions and then transferred to nitrocellulose membrane. Membranes were blocked with Odyssey blocking buffer (Li-COR) (for p24 detection) or TBS containing 5% BSA and 0.05% NaN3 (for gp120 detection) and then incubated overnight at 4°C with a sheep anti-HIV-1 gp120 polyclonal antibody (clone D7324, Aalto Bio Reagents) or for 1 h at RT with a mouse anti-HIV-1 p24 mAb (clone 749140, R&D Systems). Membranes were then incubated with the following species-specific secondary antibodies: DyLight 800-conjugated donkey Anti-Sheep IgG (Novusbio) and IRDye 800CW-conjugated goat Anti-Mouse (Li-COR) (dilution: 1/10,000). Signals were detected with a Li-COR Odyssey scanner and quantified using ImageStudioLite software. Arrow indicates gp120 bands. As control, thirty ng of purified gp120 #28 were also immunodetected. A representative experiment out of two independent experiments with distinct virus preparations is shown. B Band intensity ratios of gp120 to p24, normalized to NL4-3. Means ± SEM of two independent experiments are shown. The color code is the same as in Fig 1A. Statistics: Mann-Whitney test. (PPTX) [file ppat.1009526.s002.pptx]

## Slide 1
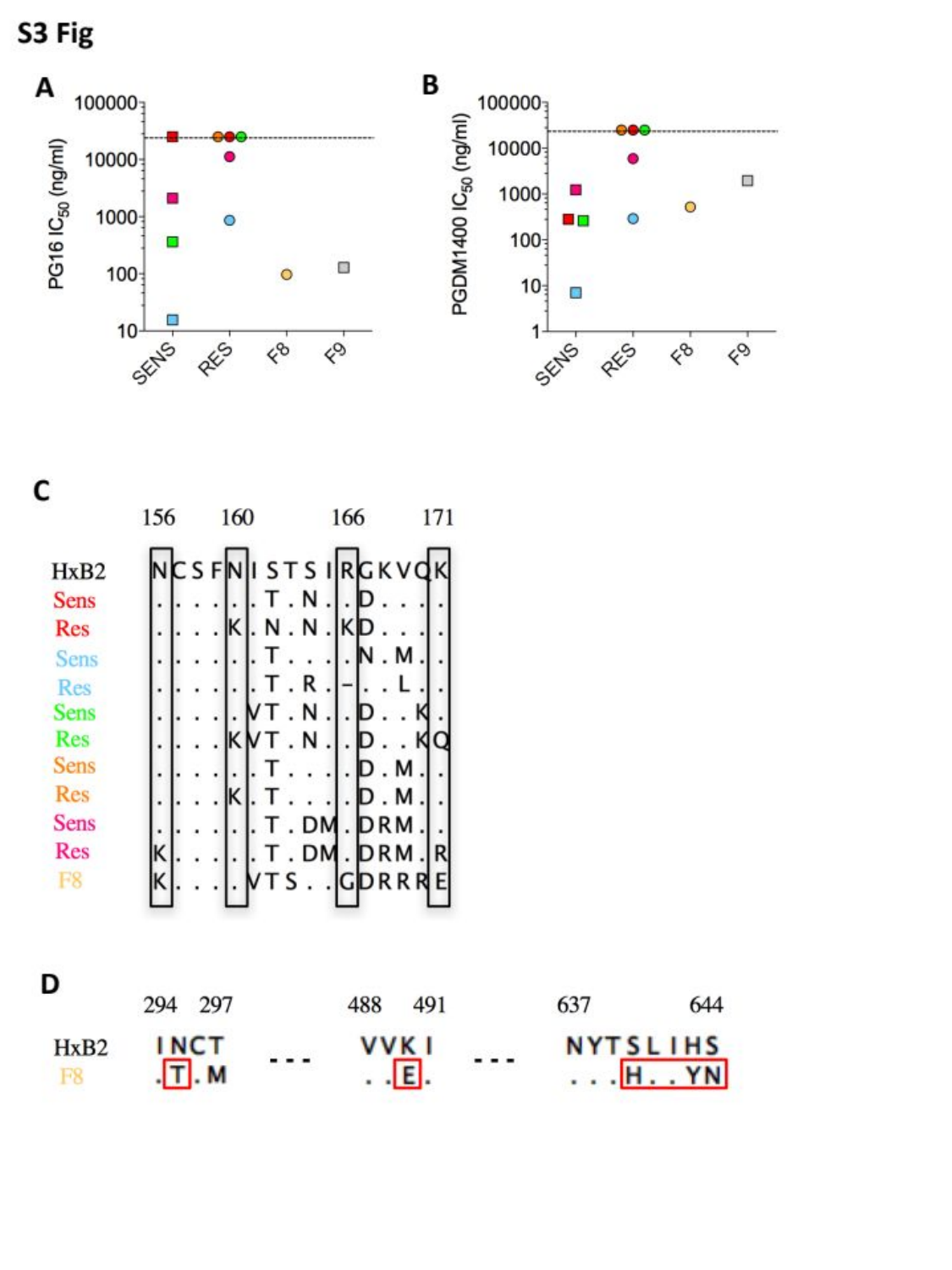

Supplement: S3 Fig — A and B Sensitivity of SENS, RES, F8 and F9 Envs to neutralization by PG16 (A) and PGDM1400 (B). Experiments were carried out and analyzed as described in Fig 3. C RES Envs, but not SENS Envs, show amino acid substitutions in the epitope for V2-apex bNAbs. The corresponding sequence in HxB2 Env is shown as reference. The color code for the patients is the same as in Fig 3. D Different residues that comprise the epitope for PGT151 at the gp120/gp41 interface are substituted in F8 Env. (PPTX) [file ppat.1009526.s003.pptx]

## Slide 1
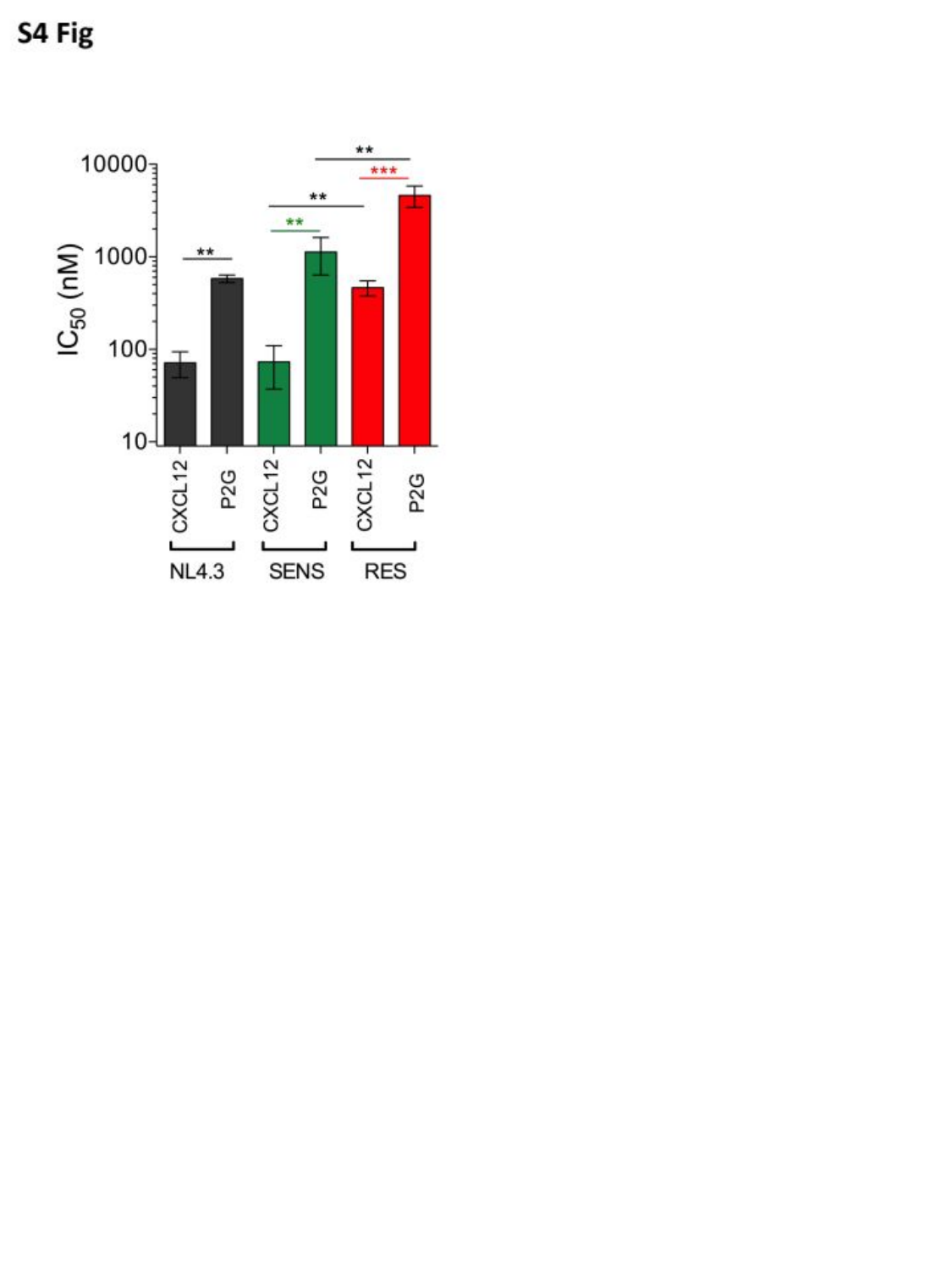

Supplement: S4 Fig — The panel shows inhibition by CXCL12 or its antagonist variant P2G of infection of primary CD4TL with viruses pseudotyped with NL4-3 Env, SENS Envs (Envs #1 and #16) or RES Envs (Envs #6 and #28). Shown are the means ± SEM of at least three independent experiments carried out with CD4TL from distinct healthy donors. *, P < .05; **, P < .01; ***, P < .001, Mann-Whitney test. (PPTX) [file ppat.1009526.s004.pptx]

## Slide 1
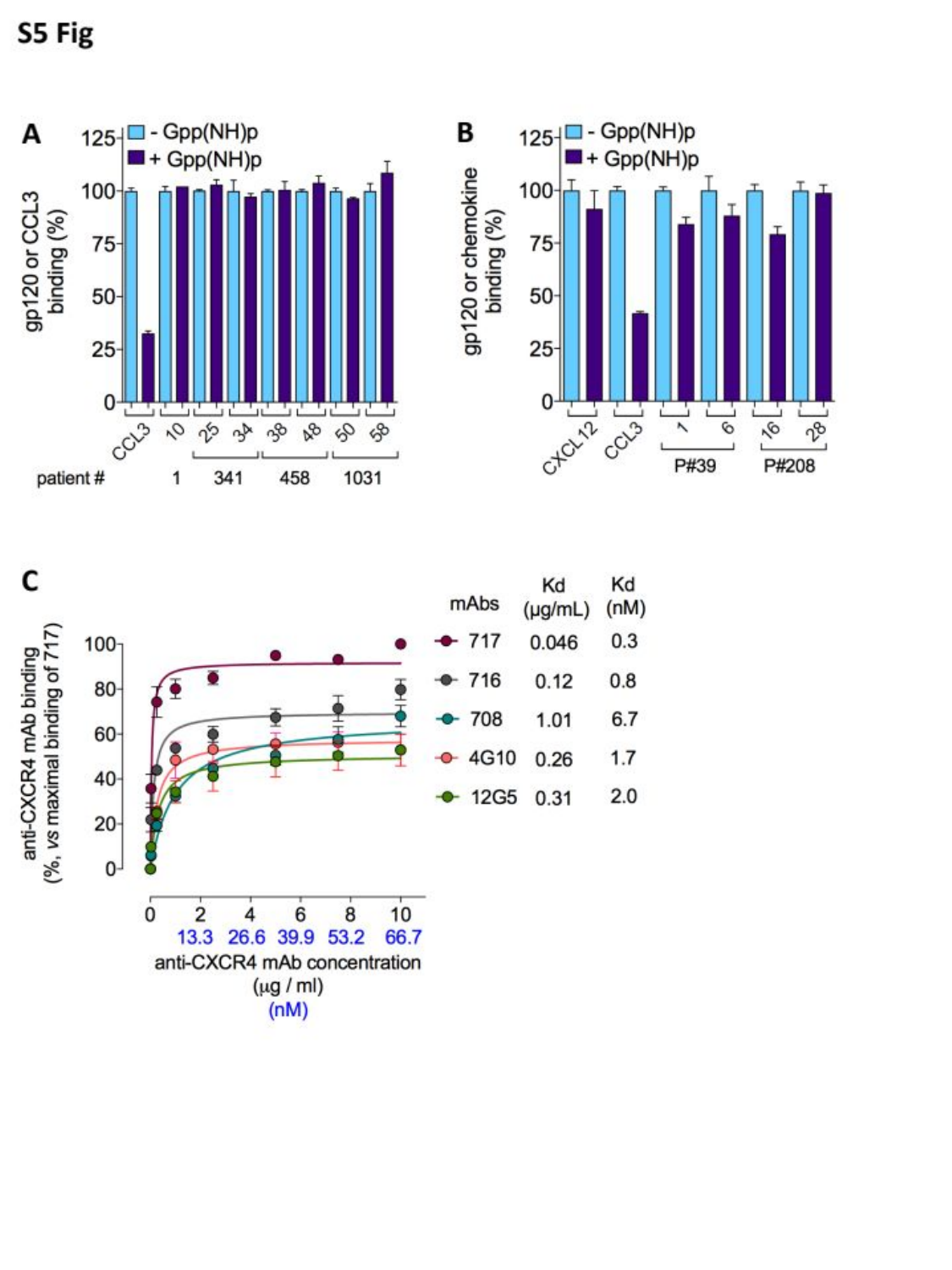

Supplement: S5 Fig — A Coupling of CCR5 to G-proteins is required for the binding of CCL3, but not for the binding of R5 gp120s. The panel represents the specific binding of 125I-CCL3 (0.1 nM) or of the R5 35S-gp120s (10 nM in complex with 30 nM sCD4) from patients P#1, #341, #458 and #1031 to membrane preparations from CCR5-expressing HEK 293T cells, in the presence or absence of 100 μM of 5’-guanylylimidodiphosphate (Gpp(NH)p), a nonhydrolysable GTP analog that uncouples permanently G proteins from receptors. Specific binding was deduced by subtracting from total binding the non-specific binding measured in the presence of 10 μM maraviroc. A representative experiment out two is shown. Results (expressed as % binding relative to binding in the absence of Gpp(NH)p) are means ± SEM of technical triplicates. B In contrast to CCL3, the binding of CXCL12 (0.5 nM) or of the X4 35S-gp120s (80 nM in complex with 300 nM sCD4) from patients P #39 and #208 to membrane preparations from PBMCs is poorly sensitive to treatment with 100 μM Gpp(NH)p. Specific binding was calculated as in panel A. Non specific binding of CXCR4-using ligands was determined with 10 μM AMD3100. Means ± SEM of two independent experiments are shown. C Saturation binding of anti-CXCR4 mAbs to CD4TL. PHA/IL-2-treated CD4TL (1 x 10^5^ cells) in PBS supplemented with 1% human serum were incubated for 2 h at room temperature with increasing concentrations of mAbs (in μg/ml and in nM) targeting the second extracellular loop (ECL2) of CXCR4 (mAbs 44 708, 44716 and 44717), its N-terminus (Nt) (4G10) or a conformational epitope encompassing ECL2, ECL3 and the Nt (12G5). Cells were then stained with an AlexaFluor 647-conjugated goat anti-mouse IgG secondary antibody for 30 min at 4°C. GMFI values for each mAb were determined by flow cytometry analysis, and subtracted of the GMFI obtained for the secondary antibody alone. Binding levels of mAbs were inferred from GMFI values and are expressed as percent of maximal binding of mAb 44717. [file ppat.1009526.s005.pptx]

## Slide 1
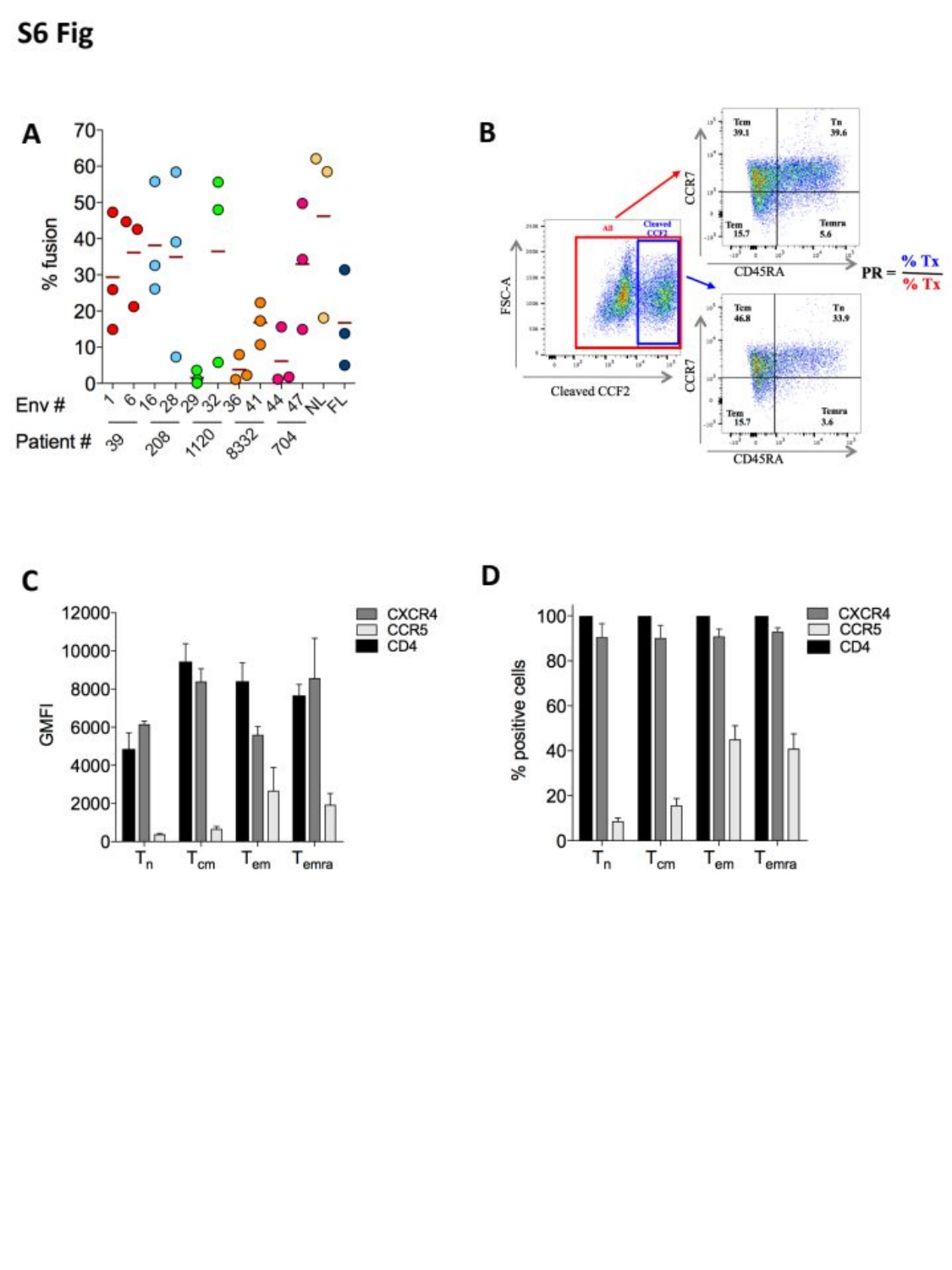

Supplement: S6 Fig — A Percent fusion of pseudotyped viruses with CD4TL. Cells (2 x 10^5^) were incubated with 100 ng Gag p24 of BLaM-vpr-containing viruses pseudotyped with either of the indicated Envs and then loaded with the BLaM substrate CCF2-AM. Percent fusion was determined by flow cytometry and represents the proportion of cells where CCF2 is cleaved by BLaM. For each virus, the mean percentages of fusion obtained from three independent experiments are plotted. B Strategy for quantifying the propensity of each CD4TL subsets to fuse with SENS and RES viruses. The different CD4TL subsets within all cells (gated in red) and within infected cells (i.e. cells positive for cleaved CCF2, gated in blue) were distinguished based on their capacity to bind anti-CD45RA and anti-CCR7 mAbs. This allowed calculating the PR value for each cell subset, which represents the ratio of its proportions in infected vs all cells. In this example, PR = 1.2 for Tcm cells, indicating that they are more abundant in infected than in uninfected cells. C Geometric Mean Fluorescence Intensity (GMFI) of CXCR4, CCR5 and CD4 mAbs on the different CD4TL subsets. CD4TL were activated for 5 days with PHA/IL-2 and then FACS-sorted prior to analysis of receptor expression levels. D Percent cells that express CXCR4, CCR5 or CD4 on each CD4TL subset. (PPTX) [file ppat.1009526.s006.pptx]

## Slide 1
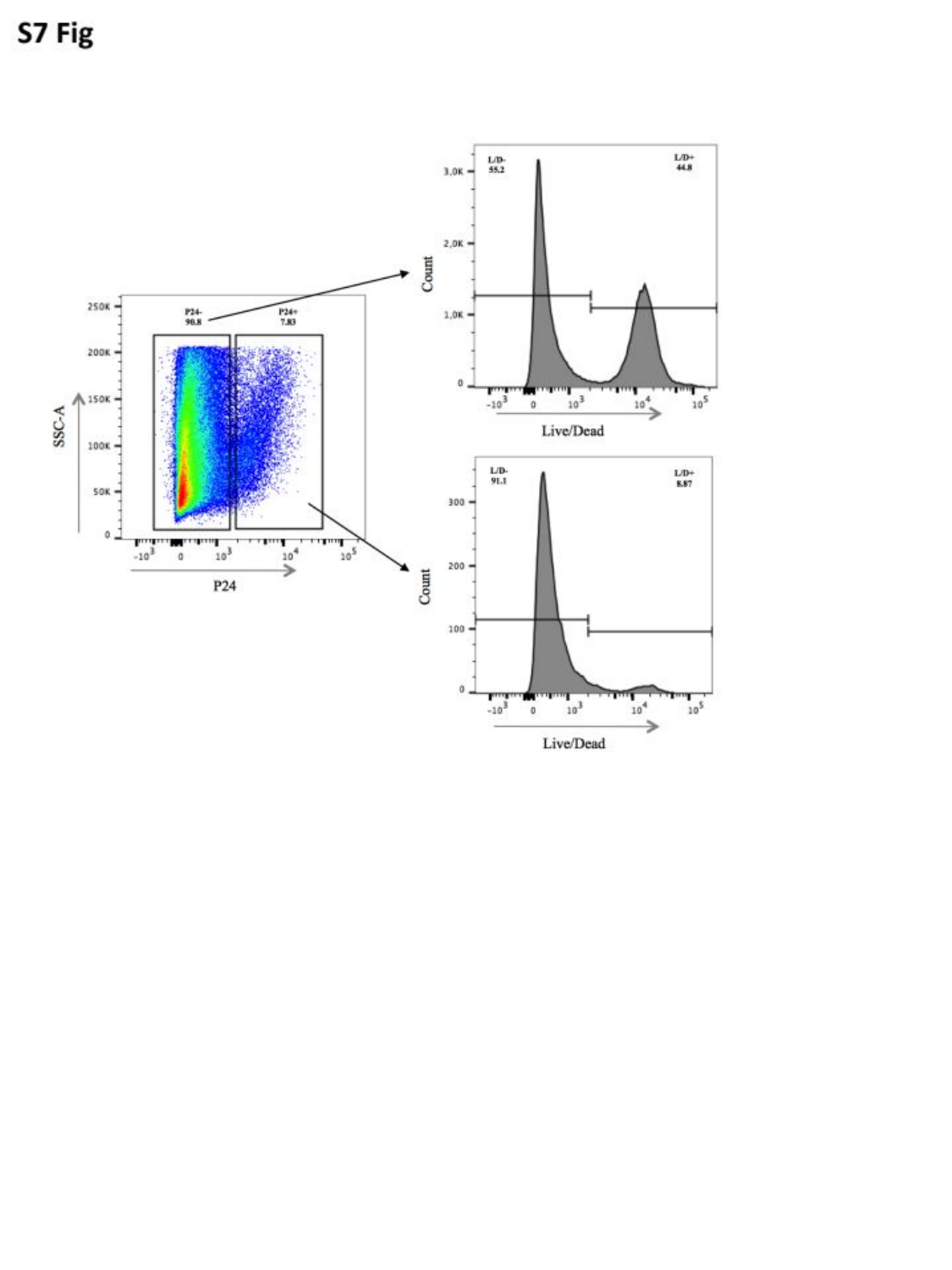

Supplement: S7 Fig — PHA/IL-2-treated CD4TL (2 x 10^5^ cells) were incubated with 50 ng p24 of SENS or RES viruses for 7 days. Productively infected (p24 positive) and bystander (p24 negative) CD4TL were then stained with the Live/Dead viability dye (L/D) and analyzed by flow cytometry. A typical experiment is shown. We measured about 10% of L/D positive cells in uninfected CD4TL cells. (PPTX) [file ppat.1009526.s007.pptx]

## Slide 1
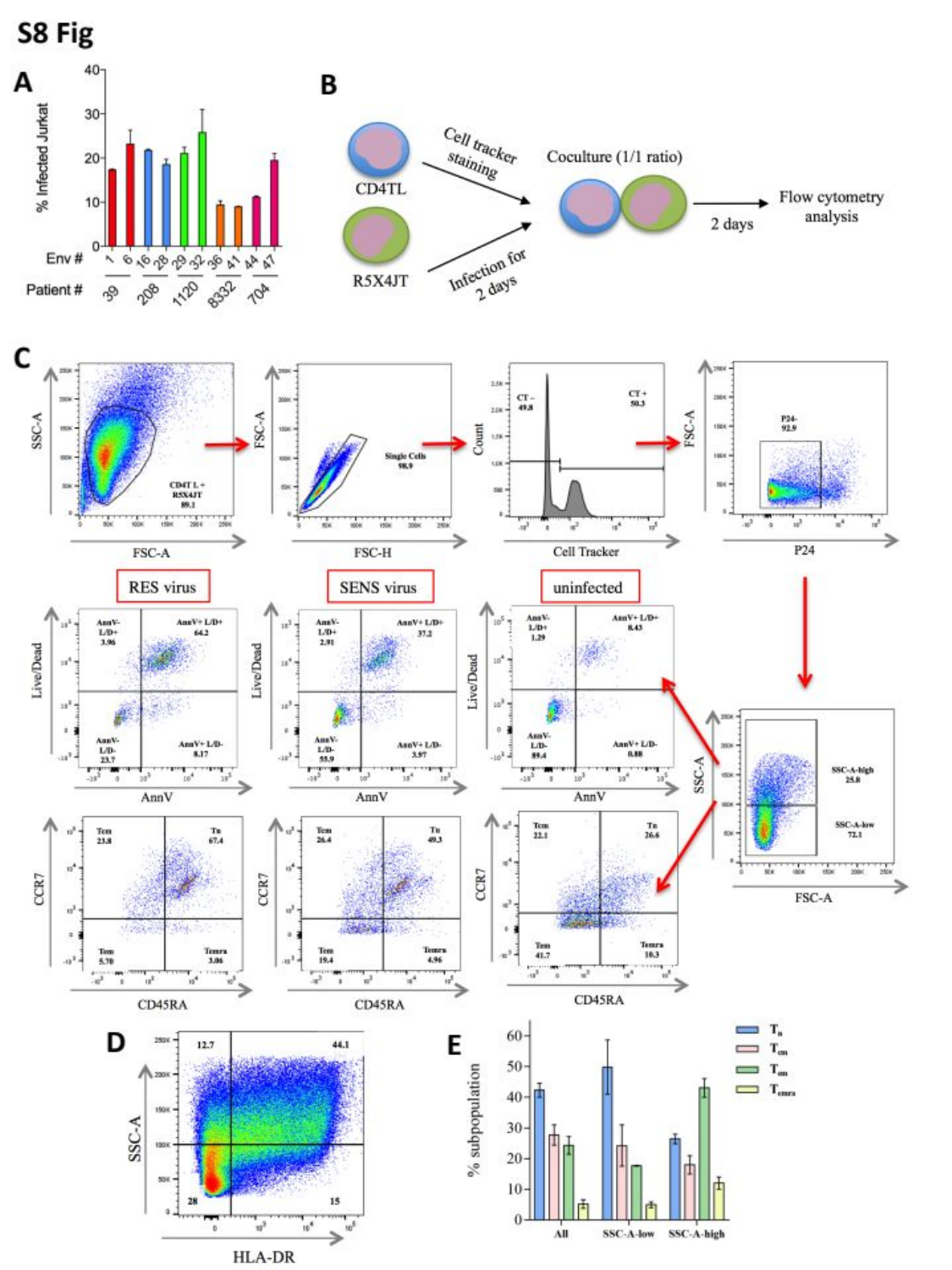

Supplement: S8 Fig — A The percentage of R5X4JT cells infected with each of the indicated viruses was determined by flow cytometry after Gag p24 staining, two days post-inoculation. B PHA/IL-2-treated CD4TL from healthy donors were stained with the CellTracker (CT) dye and then cocultured with the infected R5X4JT cells at a 1/1 ratio. Coculture experiments of CD4TL with uninfected R5X4JT cells served as control. C After two days of coculture, CT negative (R5X4JT) and positive (CD4TL) cells were stained for expression of Gag p24, CCR7 and CD45RA, apoptosis (annexinV staining) and necrosis (Live/Dead staining). Within the fraction of gag p24 negative (bystander) CD4TL (92.9%), two cell populations were distinguished according to their SSC-A and FSC-A parameters. Within the population of CD4TL with high granularity (SSC-high cells, 25.8% of total CD4TL), Tem cells (CCR7 negative, CD45RA negative) were selectively depleted in the presence of infected R5X4JT cells, but not in the presence of uninfected R5X4JT cells (41.7% of SSC-high cells). This effect was more marked in the presence of R5X4JT cells infected with RES viruses (5.7%), compared to SENS viruses (19.4%). The proportion of SSC-high CD4TL that are positive for cell death markers (annexin V and Live/Dead, upper panels) was also increased in the presence of RES viruses (64.2% of AnnV+ L/D+ double positive cells), compared to cells with SENS viruses (37.2%) and control cells (8.43%). Results from a typical experiment are shown. In comparison, viability of SSC-low CD4TL was only marginally influenced by infected R5X4JT cells. D Expression of HLA-DR was increased in SSC-high CD4TL, compared to SSC-low CD4TL. E Relative proportions of Tn, Tcm, Tem and Temra cells in PHA/IL-2-treated CD4TL (all) and its SSC-low and SSC-high subsets. SSC-high CD4TL were enriched in Tem cells, compared to SSC-low CD4TL. (PPTX) [file ppat.1009526.s008.pptx]
