# Supplementary material for: Mechanisms of HIV-1 evasion to the antiviral activity of chemokine CXCL12 indicate potential links with pathogenesis
Source: PLoS Pathog. 2021 Apr 19;17(4):e1009526. doi: 10.1371/journal.ppat.1009526 (PMC8084328; doi:10.1371/journal.ppat.1009526)
Supplement: S1 Table — (DOCX) [file ppat.1009526.s009.docx]

**S1 Table. Virological and immunological characteristics of individuals of the ACS harboring CXCR4-using viruses (Related to Fig 1A).**

| Patient | Virus clone | ID number | Tropism | CD4 T cell count (/μl of blood) | Time to SC (months) | Time to R5/X4 switch (months) |
| --- | --- | --- | --- | --- | --- | --- |
| 39 | 20.1E8 | X4-1 | X4 | 560 | 18 | 3 |
| 39 | 1.C4 | X4-6 | X4 | 10 | 50 | 36 |
| 39 | 1.F12 | X4-7 | X4 | 10 | 50 | 36 |
| 208 | 12.F4 | X4-16 | X4 | 440 | 16 | 4 |
| 208 | 4.A8 | X4-27 | nd | 10 | 76 | 65 |
| 208 | 4.A12 | X4-28 | _R5_X4^(3)^ | 10 | 76 | 65 |
| 1120^(1)^ | 11B12 | X4-29 | X4 | 270 | 53 | 7 |
| 1120^(1)^ | 14G11 | X4-32 | X4 | 10 | 66 | 20 |
| 8332^(2)^ | 21B3 | X4-36 | X4 | 300 | 34 | 4 |
| 8332^(2)^ | 63A8 | X4-41 | X4 | 50 | 78 | 49 |
| 704 | 13A5 | X4-44 | _R5_X4^(3)^ | 610 | 61 | 3 |
| 704 | 16D6 | X4-47 | R5X4^(4)^ | 210 | 70 | 12 |

^(1)^ Patient #1120 initiated zidovudine (azidothymidine, AZT) monotherapy 21 months after seroconversion (SC); ^(2)^ Patient #8832 initiated AZT monotherapy 34 months after SC (*i.e.* at the time of isolation of Env #36). ^(3)(4)^ These viruses showed residual usage of CCR5 in U87-CD4-CCR5 cells (< 1 %^(3)^ and 7 %^(4)^, compared to the prototypic R5 strain JR-FL).

nd, not determined
