# Supplementary material for: Mechanisms of HIV-1 evasion to the antiviral activity of chemokine CXCL12 indicate potential links with pathogenesis
Source: PLoS Pathog. 2021 Apr 19;17(4):e1009526. doi: 10.1371/journal.ppat.1009526 (PMC8084328; doi:10.1371/journal.ppat.1009526)
Supplement: S2 Table — (DOCX) [file ppat.1009526.s010.docx]

**S2 Table. Virological and immunological characteristics of individuals of the ACS harboring R5 viruses (Related to Fig 1B).**

| Patient | Virus clone | ID number | Tropism | CD4 T cell count (/μl of blood) | Time to SC (months) |
| --- | --- | --- | --- | --- | --- |
| 1 | H1.15 3A7 | R5-1 | R5 | 1050 | 26 |
| 1 | H1.15 3B5 | R5-2 | R5 | 1050 | 26 |
| 1 | H1.15 3B10 | R5-3 | R5 | 1050 | 26 |
| 1 | H1.46 5B1 | R5-10 | R5 | 190 | 107 |
| 1 | H1.46 5B3 | R5-11 | R5 | 190 | 107 |
| 1 | H1.46 5B6 | R5-12 | R5 | 190 | 107 |
| 232 | H4.49 7A12 | R5-13 | R5 | 540 | 36 |
| 232 | H4.137 2G1 | R5-22 | R5 | 150 | 127 |
| 341 | H5.14 5C6 | R5-25 | R5 | 630 | 30 |
| 341 | H5.14 5D5 | R5-26 | R5 | 630 | 30 |
| 341 | H5.14 6B12 | R5-27 | R5 | 630 | 30 |
| 341 | H5.75 6C4 | R5-34 | R5 | 90 | 128 |
| 341 | H5.75 7A9 | R5-35 | R5 | 90 | 128 |
| 341 | H5.75 7E9 | R5-36 | R5 | 90 | 128 |
| 458 | H2.12 B11 | R5-37 | R5 | 350 | 20 |
| 458 | H2.12 C12 | R5-38 | R5 | 350 | 20 |
| 458 | H2.12 D6 | R5-39 | R5 | 350 | 20 |
| 458 | H2.43 5C1 | R5-46 | R5 | 60 | 86 |
| 458 | H2.43 5C8 | R5-47 | R5 | 60 | 86 |
| 458 | H2.43 5D3 | R5-48 | R5 | 60 | 86 |
| 1031 | H3.20 8A12 | R5-49 | R5 | 520 | 22 |
| 1031 | H3.20 8C1 | R5-50 | R5 | 520 | 22 |
| 1031 | H3.20 8F1 | R5-51 | R5 | 520 | 22 |
| 1031 | H3.65 9D8 | R5-58 | R5 | 50 | 91 |
| 1031 | H3.65 9E7 | R5-59 | R5 | 50 | 91 |
| 1031 | H3.65 9H2 | R5-60 | R5 | 50 | 91 |
