# Supplementary material for: Mechanisms of HIV-1 evasion to the antiviral activity of chemokine CXCL12 indicate potential links with pathogenesis
Source: PLoS Pathog. 2021 Apr 19;17(4):e1009526. doi: 10.1371/journal.ppat.1009526 (PMC8084328; doi:10.1371/journal.ppat.1009526)
Supplement: S3 Table — (DOCX) [file ppat.1009526.s011.docx]

**S3 Table. CD4TL count, viral load and viral tropism in the patients who were diagnosed with HIV-1 at a chronic or advanced stage of infection (Related to Figs 1C, S1E and S1F).**

| Patient | Tropism | Viral load log 10 copies/ml) | CD4 T cell count (/μl of blood) |
| --- | --- | --- | --- |
| HEAX^(1)^ | R5X4 | 5.3 | 8 |
| INCE^(1)^ | R5X4 | 5.1 | 2 |
| TEOT^(1)^ | R5X4 | 4.6 | 7 |
| ZEYB^(1)^ | X4 | 5 | 9 |
| OXHU^(1)^ | R5X4 | 5.7 | 8 |
| EBTY^(1)^ | X4 | 4.9 | 45 |
| OSQA^(1)^ | R5X4 | 5.4 | 146 |
| IFQA^(1)^ | R5X4 | 5.3 | 140 |
| PECJAN^(2)^ | X4 | 4.9 | 26 |
| PONMIC^(2)^ | R5X4 | 5.4 | 496 |
| DIAAY^(2)^ | R5X4 | 4.3 | 678 |
| BART^(2)^ | R5X4 | 4.8 | 383 |
| DINA^(2)^ | R5X4 | 5.7 | 354 |

^(1)^ Patients enrolled in the ANRS 146 OPTIMAL clinical trial; ^(2)^ Patients attending the Department of Infectious Diseases of Toulouse University Hospital.
