# Supplementary material for: Mechanisms of HIV-1 evasion to the antiviral activity of chemokine CXCL12 indicate potential links with pathogenesis
Source: PLoS Pathog. 2021 Apr 19;17(4):e1009526. doi: 10.1371/journal.ppat.1009526 (PMC8084328; doi:10.1371/journal.ppat.1009526)
Supplement: S4 Table — (DOCX) [file ppat.1009526.s012.docx]

**S4 Table. Baseline characteristics of patients diagnosed at the time of PHI (Related to Figs 1D, S1C, S1D, S1G and S1H).**

| Patient | Tropism | Fiebig stage | Gender | Transmission route | Viral load (log10 copies/ml) | CD4 T cell count (/μl of blood) |
| --- | --- | --- | --- | --- | --- | --- |
| F3^(1)^ | R5X4 | II-III | Female | IDU | 5.9 | 457 |
| F5^(1)^ | R5X4 | V | Male | IDU | 5.6 | 939 |
| F7^(1)^ | R5X4 | IV-V | Male | MSM | 3.8 | 411 |
| F8^(1)^ | R5X4 | V | Male | MSM | 5.4 | 45 |
| F9^(1)^ | R5X4 | IV-V | Male | MSM | 4 | 539 |
| 2.2^(2)^ | R5X4 | II-III | unk | unk | > 7 | 794 |
| 19.1^(2)^ | X4 | II-III | unk | unk | 5.1^*^ | 268^*^ |
| 6.1^(2)^ | R5 | II-III | unk | unk | > 7 | 553 |
| 11.1^(2)^ | R5 | II-III | unk | unk | 6.1^*^ | 883^*^ |
| 13.1^(2)^ | R5 | II-III | unk | unk | 5^*^ | 676^*^ |
| 14.1^(2)^ | R5 | II-III | unk | unk | 4.8^*^ | 1730^*^ |
| 15.1^(2)^ | R5 | II-III | unk | unk | 5.4 | nd |
| 17.1^(2)^ | R5 | II-III | unk | unk | 6.2^*^ | 431^*^ |
| 22.1^(2)^ | R5 | II-III | unk | unk | 3.4 | nd |
| 23.1^(2)^ | R5 | II-III | unk | unk | 4.8 | 388^*^ |
| 25.1^(2)^ | R5 | II-III | unk | unk | 4.2 | nd |

The patients were diagnosed ^(1)^ at the Toulouse University Hospital or ^(2)^ at the Sandoval Health Center. Patients F3, F5 and F7 have previously been described as Patients #87, #15 and #93 in Raymond et al., AIDS, 2010;24(15):2305-12. ^(*)^ Data were obtained 1-3 months after the sample used in the present study was drawn. IDU, intravenous drug user; MSM, men who have sex with men; unk, unknown; nd, not determined.
