# Supplementary material for: Mechanisms of HIV-1 evasion to the antiviral activity of chemokine CXCL12 indicate potential links with pathogenesis
Source: PLoS Pathog. 2021 Apr 19;17(4):e1009526. doi: 10.1371/journal.ppat.1009526 (PMC8084328; doi:10.1371/journal.ppat.1009526)
Supplement: Attachment — Submitted filename: review plospath 2021.docx [file ppat.1009526.s013.docx]

Armani-Tourret et al., describe extensively the discovery of HIV-1 isolates that are found to less sensitive to the chemokine CXCL12 and these viruses are highly pathogenic.

The work is well performed, well discussed and planned and many and different assays are done and performed with great expertise.

Some comments :

Page 2 : Abstract, last sentence :

“…but also sheds new light into antiviral strategies targeting CXCR4..”,

It is not clear how this can be interpretated from these presented data?

Page 4: “In vitro, CXCL12 inhibits viral entry by preventing Env binding to CXCR4 and triggering endocytosis of the receptor”.

In the past it was shown that endocytosis is the main mechanism of antiviral action of CXCL-12 and not just binding and affinity to its receptor.

The effect of endocytosis -or the absence of- is not studied in the different cellsystems to explain a difference in the infection of the SENS or RES viruses.

Page 6:

“X4-tropic”, better to use X4 or CXCR4-tropic or CXCR4-using.

Page 6:

“In these severe CD4-lymphopenic patients, however, we found no correlation between the degree of resistance

to CXCL12 and the CD4TL count (**S1F Fig**).”

So not clear what this then means the CXCL12 resistance and the link with the low CD4TL count?

Page 7:

“Late X4 Envs were three-fold less sensitive to inhibition by sCD4 than early Envs (IC50 = 56 *vs* 146 nM, *P* = 0.016).”.

So this could mean a “general” phenomenon and that these viruses use a less amount of receptors (CD4 and CXCR4) on the cell membrane for infection.?

Page 9:

“Furthermore, RESgp120s were only partially inhibited at the highest CXCL12 concentration tested (1 μM), suggesting that they also interact with a fraction of CXCR4 that does not bind CXCL12 at all.”

Can this be then that this fraction of CXCR4 is not internalized by CXCL-12 and thus can be used by HIV to enter the cells? Is there also a potential effect on dimerization of CXCR4?

Page 10:

Binding of 4G10 to high- and low-CXCL12 affinity receptors may explain why the mAb inhibits as efficiently SENS and RES viruses (Fig. 4D).

But still there is ~40% of infection for both viruses in the presence of this mAb?

Is there any role of CXCR7 in the antiviral assays as this receptor also (strongly) binds CXCL-12?

And thus there is less CXCL-12 available to bind to CXCR4?

Page 10:

“R5-tropic”, better to use R5 or CCR5-tropic or CCR5-using.

Page 14:

“… suggest that binding to CXCR4 molecules not accessible to CXCL12 is the reason why mAb 4G10 efficiently inhibits RES viruses.”

What epitope is 4G10 recognizing on CXCR4? Would also be useful to mention this for the other mabs used in this study.
