# Supplementary material for: Mechanisms of HIV-1 evasion to the antiviral activity of chemokine CXCL12 indicate potential links with pathogenesis
Source: PLoS Pathog. 2021 Apr 19;17(4):e1009526. doi: 10.1371/journal.ppat.1009526 (PMC8084328; doi:10.1371/journal.ppat.1009526)
Supplement: Attachment — Submitted filename: Response to reviewers Armani et al., PP-R1 - copie.docx [file ppat.1009526.s014.docx]

March 24^th^ 2021

Dr Bernard Lagane, PhD,

Toulouse Institute for Infectious

and Inflammatory Diseases (INFINITy),

INSERM Unit U1291 (ex U1043),

BP 3028,

31024 Toulouse CEDEX 03, France

Phone: +33562748385

e-mail: [bernard.lagane@inserm.fr](mailto:bernard.lagane@inserm.fr)

**Armani-Tourret et al., The HIV-1 strains that evade the antiviral activity of the chemokine CXCL12 are highly pathogenic, PPATHOGENS-D-20-02505**

**Response to Reviewers**

**Reviewer #1**

We thank the reviewer for the positive and insightful comments on our work. All the issues that were raised are discussed below and new data have been included in the revised manuscript.

**I think the authors are addressing an interesting topic in a way that others have not, and they have made considerable progress. I struggled a bit in assimilating this study as it seems to me that there are two very worthy goals. The first goal is to determine if CXCL12 resistant viruses are linked to accelerated disease progression, and for this you need numbers: a reasonable number of X4 viruses need to be examined from different patients to see if there is in fact a correlation. It is here that the study falls short in that only a very small number of patients and Envs were examined – the results certainly suggest that there may be a correlation, but looking at only 5 early stage and 5 late stage patients in my opinion does not justify the conclusion stated in the title of the paper, and it is this goal that makes the study appropriate for a more general audience.**

Actually, the title reflects two main observations we report in the paper, first, the presence of CXCL12-resistant viruses, which we only observed in patients with low CD4 T cell counts, and secondly, the greater ability of five CXCL12-resistant viruses, used as models, to kill bystander CD4 T cells. We acknowledge that while this latter observation suggests that there could be a causal link between the presence of RES viruses and CD4 T cell depletion, further clinical and immunological data on a larger number of patients (*e.g.* cohorts of patients with progressive infection with or without CXCL12-resistant viruses) would be required to determine whether this is indeed the case. In this aspect, our statement of the increased pathogenicity of RES viruses should be viewed more as a proof-of-principle that invites future research. The fact that RES viruses *co-occur* with low levels of CD4 T cells is nonetheless based on analysis of a greater amount of viruses (about thirty CXCR4-using viruses from three different cohorts have been tested for their sensitivity to CXCL12). It is thus more than likely that there is *an association* between RES viruses and CD4 T cell depletion, regardless of whether these viruses are the “cause” or the “consequence” of CD4 T cell depletion.

Because the reviewer mentions that the title goes somewhat beyond the conclusions of our work, we can propose the following one that perhaps summarizes more of our data and puts less emphasis on the causal link between RES viruses and increased pathogenicity: “Analysis of HIV-1 strains that evade the antiviral activity of the chemokine CXCL12: Involved mechanisms and links with pathogenesis”. We can replace the current title with this new one if the reviewer finds it more suitable. Alternatively, we would be willing to consider other titles that may be suggested by the reviewer.

**A few technical points:**

**I think the study relies only on virus pseudotype assays for infections, which raises the possibility that differences in Env expression and virus incorporation could influence some of the results. It would be too painful to generate replication competent viruses in sufficient numbers, so are the authors reasonably confident that the results are not confounded by Env incorporation differences?**

Data in the manuscript suggest that if indeed differences exist in the degree of Env incorporation between SENS and RES viruses, they do not influence sensitivity to CXCL12. If this were the case, one would expect sensitivity to CXCL12 to vary in the same way as sensitivity to other entry inhibitors targeting CD4 or CXCR4. Actually, this is not what we observed. For instance, SENS and RES viruses are equally sensitive to the anti-CD4 mAb Q4120 (**Fig 2B**) and to the anti-CXCR4 mAb 4G10 (**Fig 4D and 4E**). Similarly, we did not necessarily observe cross-resistance of RES viruses to AMD3100. In the same line, there was no correlation between infectivity of viruses and their capacity to resist CXCL12. Finally, RES and SENS viruses were also equally sensitive to some bNAbs targeting Env (VRC01, 10E8 and PGT121).

The reviewer’s comment remains nonetheless very relevant. We therefore examined Env expression on the different viruses pseudotyped with Envs of the Amsterdam cohort or NL4-3 Env as control. This was done by western blot analysis of gp120 and p24 expression into the viruses. This new experiment is now presented as **S2 Fig** in the revised version of the manuscript. As speculated by the reviewer, there is some degree of variability in the amount of gp120 from one virus to another. It is however clearly apparent that there is no correlation between expression of gp120 and sensitivity to inhibition by CXCL12. Considering these new data with the previous ones, we conclude that Env incorporation differences are very unlikely to influence the results presented in our manuscript.

**Some nice competition binding assays are performed, and one of the conclusions is that resistant gp120 proteins bind to forms of CXCR4 that sensitive gp120 proteins do not. If this is correct, then would it not follow that sensitive gp120 proteiåns could not prevent resistant gp120 proteins from binding to CXCR4?**

Our experiments are indeed consistent with the view that some of the CXCR4 molecules to which RES gp120s bind are not recognized by SENS gp120s, and *vice versa*. The reviewer proposes an alternative approach to illustrate this that is interesting. We indeed previously performed competition binding experiments of ^35^S-gp120s by unlabeled gp120s to determine that distinct R5 Envs bind differentially to distinct CCR5 populations (Colin et al., 2018, PLoS Pathog). However, there are technical limitations to these approaches in the case of X4 Envs due to their very low affinity for CXCR4. A complete displacement of bound ^35^S-gp120 (usually used at a concentration close to the Kd) would require the addition of huge amounts of unlabeled gp120 (in the μM range), which raises two issues. First, the purification yield of gp120s does not allow us to use gp120 concentrations that exceed few hundreds of nM in the binding experiments.

Second, the binding experiments are performed under conditions where gp120/soluble CD4 complexes are preformed, therefore avoiding binding of gp120s to membrane CD4 (see our comments on this point in the “Materials and methods” section). For that purpose, concentrations of sCD4 are used in excess, which might be difficult to achieve under conditions of very high concentrations of gp120s.

With these limitations in mind, we still evaluated the reviewer’s hypothesis and measured the binding of ^35^S-labeled gp120#28 (RES gp120) to crude membranes from unstimulated PBMCs in the presence of cold gp120#28 or gp120#16 (SENS gp120). To favor displacement, we used ^35^S-gp120#28 at a very low concentration, *i.e.* 15 nM, and the highest possible concentrations for the unlabeled gp120s (590 nM and 346 nM for gp120 #16 and #28, respectively). Soluble CD4 was used à 1.9 μM. As shown in the accompanying figure, both cold gp120s displaced the binding of ^35^S-gp120#28, but cold gp120#28 did so with greater efficacy. These results are particularly striking if one consider that gp120#28 was used at a lower concentration and was found to bind fewer receptors in the saturation binding assays (**Fig 4A**), compared to gp120#16. These results suggest that while some CXCR4 molecules are shared by both gp120s, others that bind gp120#28 are poorly accessible to gp120#16. They are therefore consistent with our other data in the paper that differences exist between SENS and RES gp120s in the nature of the CXCR4 molecules to which they bind. It should be noted that this new experiment should however be interpreted with caution because ^35^S-gp120 #28 was used at a very low concentration (at this concentration, only 10-15 % of receptors are bound by the protein) and due to insufficient amounts of purified gp120s, we were unable to test other concentrations of unlabeled gp120s. It is also not known whether the distinct receptors that differentially bind SENS and RES gp120s represent *independent* or *dependent* receptor populations. Independent low-CXCL12 and high-CXCL12 affinity receptors that reside in distinct membrane domains have been described (Nguyen & Taub, 2002, JI). But it is also well-known that CXCR4 exists not only as monomers but also forms homodimers or higher-size oligomers as well as nanoclusters (Wu et al., Science, 2010; Armando et al., 2014, FASEB J; Martinez-Munoz et al., 2018, Mol Cell and our results Lagane et al., Blood, 2008) and previous data have shown that receptors with high and low affinity for CXCL12 may coexist within CXCR4 oligomers (Sohy et al., 2007, JBC). It is believed that the binding of ligands to one protomer may transmit conformational changes to neighboring protomers, thereby changing their ligand binding properties (Kufareva et al., 2014, PNAS). In this context, it is quite possible that a gp120 (e.g. gp120 #16) may influence the binding of another gp120 (e.g. gp120#28), even though these two gp120s do not bind to the same receptors.

**R1 Fig. Competition binding experiments of ^35^S-gp120 #28 by unlabeled gp120s #16 or #28.** Results are expressed relative to binding in the absence of competitor (100 %, B_0_).

**Reviewer #2**

We thank the reviewer for the positive evaluation of our work and the constructive comments, which are addressed below and have been taken into account in the revised manuscript.

**Some comments :**

**Page 2 : Abstract, last sentence :**

**“…but also sheds new light into antiviral strategies targeting CXCR4..”,**

**It is not clear how this can be interpretated from these presented data?**

In response to the reviewer’s comment, the conclusion has been clarified and is now further oriented in light of our findings. The discussion on page 14, which the last sentence of the abstract refers to, has also been rephrased to better describe the therapeutic perspectives of our work.

**Page 4: “In vitro, CXCL12 inhibits viral entry by preventing Env binding to CXCR4 and triggering endocytosis of the receptor”.**

**In the past it was shown that endocytosis is the main mechanism of antiviral action of CXCL-12 and not just binding and affinity to its receptor.**

**The effect of endocytosis -or the absence of- is not studied in the different cellsystems to explain a difference in the infection of the SENS or RES viruses.**

To address this point, we repeated infection experiments of primary CD4 T lymphocytes with SENS viruses, RES viruses or the prototypic CXCL12-sensitive strain NL4-3, in the presence of varying concentrations of wild-type CXCL12 or the CXCL12 mutant P2G. This mutant binds CXCR4 with similar affinity compared to the wild-type chemokine, but does not induce receptor endocytosis (Crump et al., EMBO J, 1997; Tilton et al., JEM, 2000; Jaracz-Ros et al., JLB, 2020). The comparison between P2G and CXCL12 therefore allowed us to investigate whether the decreased ability of CXCL12 to inhibit RES viruses is due to a decrease in its binding to CXCR4 or in its ability to downregulate the receptor.

As shown in new **S4 Fig** of the revised manuscript, P2G inhibited infection with NL4-3 with a 8-fold reduced potency compared to CXCL12 (IC_50_ = 72 *vs* 543 nM, *P* = 0.0043). Our data are similar to those reported in Crump et al., 1997 (IC_50_ = 79 *vs* 562 nM). The decreased antiviral potency of P2G likely reflects its inability to downregulate CXCR4. Indeed, previous work reported that preventing CXCR4 downregulation decreases the anti-HIV-1 potency of CXCL12 by a similar order of magnitude (in the range of 10-fold in Amara et al., JEM, 1997). In new **S4 Fig**, P2G shows a comparable reduced potency to inhibit SENS (15-fold lower) and RES (10-fold) viruses, compared to CXCL12.

These data first indicate that receptor endocytosis contributes to the inhibitory effects of CXCL12 against both SENS and RES viruses. They also indicate that similarly to CXCL12, P2G inhibits RES viruses less potently than SENS viruses. Interestingly, the magnitude to which the antiviral potencies are reduced is in the same range for both chemokines (roughly 5-fold in this series of experiments). This strongly suggests that the reduced potency of CXCL12 to inhibit RES viruses originates from its decreased binding to CXCR4, and not to downstream defects in the capacity of receptors to be internalized (it is however reasonable to assume that the reduced affinity of CXCL12 for certain receptors may result in a decrease in its capacity to internalize these receptors). These data are actually consistent with our results in **Fig 4B and 4C**, showing that CXCL12 has a lower affinity for receptors that bind RES gp120s.

These experiments are presented in new **S4 Fig** and are described in a new paragraph on pages 8/9 of the revised manuscript.

**Page 6:**

**“X4-tropic”, better to use X4 or CXCR4-tropic or CXCR4-using.**

We have modified the text on page 6 as requested by the reviewer.

**Page 6:**

**“In these severe CD4-lymphopenic patients, however, we found no correlation between the degree of resistance**

**to CXCL12 and the CD4TL count (S1F Fig).”**

**So not clear what this then means the CXCL12 resistance and the link with the low CD4TL count?**

We agree with the reviewer that this sentence may be confusing. It has therefore been rephrased in the new version of the manuscript (on page 6). Here, we indeed show that CXCL12 resistant viruses are frequent and almost exclusively present in patients with low CD4TL count, and that RES viruses likely accelerate CD4TL depletion. However, as illustrated in **S1F Fig**, which the above-mentioned sentence referred to, CD4TL depletion in the late stage of infection can also occur in the absence of RES viruses. This was actually expected because in the natural course of infection, CD4TL depletion can occur regardless of viral tropism. This is why in late diagnosed patients with low CD4TL counts (i.e. patients from the OPTIMAL clinical trial in the manuscript), no correlation can be observed between the degree of resistance to CXCL12 and the CD4TL counts. This lack of correlation simply indicates that other mechanisms than the presence of RES viruses can contribute to CD4TL depletion in patients infected with CXCR4-using viruses.

**Page 7:**

**“Late X4 Envs were three-fold less sensitive to inhibition by sCD4 than early Envs (IC50 = 56 *vs* 146 nM, *P* = 0.016).”.**

**So this could mean a “general” phenomenon and that these viruses use a less amount of receptors (CD4 and CXCR4) on the cell membrane for infection.?**

As noted by the reviewer, our observation that late and early Envs are differentially sensitive to sCD4 suggest that they may have differential requirements for receptor expression levels. In particular, the increased resistance of late Envs suggests that they adopt a more closed conformation compared to early Envs and require more receptors for entry. To test this hypothesis, we compared late and early Envs in infection assays of 293-Affinofile cells in which we stably expressed high or low levels of CXCR4 and then induced or not expression of CD4 using minocycline, as previously described (Johnston, JVI, 2009) (See panels A and B of **R1 Fig** attached below). In the absence of minocycline, we previously showed that Affinofile cells express minimal amount of CD4, *i.e.* in the range of 4,000 molecules per cell (Colin et al., 2018). Under these conditions of low CD4 expression levels, we (Colin et al., PLoS Pathog, 2018) and others (Joseph et al., JVI, 2014) showed that R5 Envs that adopt an open conformation, but not those that are in a closed conformation, can infect Affinofile cells.

In contrast however, as shown in panel C of **R1 Fig**, we found that all X4 Envs infected only marginally 293-Affinofile cells under conditions of low CD4 expression level, regardless of their sensitivity to sCD4 and the amount of CXCR4 at the cell surface. These findings illustrate that X4 Envs are more dependent on the level of CD4 than on that of CXCR4. This can be explained by the fact that X4 Envs have substantially lower affinity for CXCR4 (K_D_ > 100 nM, see **Fig 4A**) than for CD4 (in the nM range, see **Fig 2A**). Of note, R5 Envs show a generally higher affinity for CCR5 (K_D_ ~ 10 nM, Colin et al., PLoS Pathog, 2018), which explains why they are less dependent on CD4 levels than X4 Envs and why when they adopt an open conformation, unlike X4 Envs, they are able to infect 293-Affinofile cells with low levels of CD4.

In conclusion, we show here that regardless of their degree of opening and their sensitivity to sCD4, X4 Envs require high levels of CD4 for viral entry, presumably due to low affinity for CXCR4.

**R1 Fig. Differences in the sensitivity of X4 Envs to sCD4 do not translate into differential requirements for CD4 expression. A.** Expression of CD4 at the surface of Affinofile cells expressing low (green curves) or high (red curves) levels of CXCR4, stimulated (GMFI = 6951 and 4689, respectively) or not (GMFI = 235 and 179, respectively) with minocycline. **B.** Expression of CXCR4 on Affinofile cells expressing low (GMFI = 153) or high levels (GMFI = 11033) of the receptor. Expression of CXCR4 did not change after stimulation with minocycline. **C.** Affinofile cells expressing low or high levels of CXCR4, and stimulated or not with minocyline were infected with viruses pseudotyped with early Envs (E, # 1, #16 and #29) or late Envs (L, #6, #28, #32). Infectivity of viruses was determined in the cell lysates by measuring luciferase activity (RLU).

**Page 9:**

**“Furthermore, RESgp120s were only partially inhibited at the highest CXCL12 concentration tested (1 μM), suggesting that they also interact with a fraction of CXCR4 that does not bind CXCL12 at all.”**

**Can this be then that this fraction of CXCR4 is not internalized by CXCL-12 and thus can be used by HIV to enter the cells? Is there also a potential effect on dimerization of CXCR4?**

As noted by the reviewer, the possibility exists that the receptors that do not bind CXCL12 represent an *independent* receptor population that is refractory to endocytosis. This could be the case if, for instance, low- and high-CXCL12 affinity receptors reside in distinct membrane domains, as previously suggested (Nguyen & Taub, 2002, JI). If so, the receptors that do not bind CXCL12 could indeed contribute to viral entry to some extent even in the presence of high concentrations of the chemokine. Of note, the competition experiments in **Fig 4B and C** suggest that in addition to these receptors, RES gp120s interact with multiple CXCR4 forms which, on the whole, exhibit lower affinity for CXCL12 than the receptors to which SENS viruses bind. These low affinity receptors, and not just the receptors that do not bind CXCL12, could contribute to resistance to CXCL12. To clarify this point, we have rephrased the title of the paragraph on page 9 as follows: “RES viruses use CXCR4 receptors with low affinity for CXCL12.”

As discussed above, new **S4 Fig**, however, clearly shows that receptor endocytosis contributes to inhibition of RES viruses by CXCL12. This suggests that to some extent, the receptors to which RES viruses bind that are of low affinity for the chemokine, or even those that do not bind it at all, are internalized in response to CXCL12. Actually, low affinity interactions may be sufficient for chemokines to induce receptor endocytosis (see our previous work, Colin et al., 2013, PNAS). An additional explanation to the above paradox would be that high-CXCL12 and low-CXCL12 affinity receptors form receptor populations *that are dependent on each other*. High- and low-CXCL12 affinity receptors could form two distinct receptor populations that are in equilibrium with each other, so that the disappearance of one population (the high-affinity population) after endocytosis eventually leads to endocytosis of the other. Alternatively, but not exclusively, it is known that CXCR4 exists not only as monomers but also forms homodimers or higher-size oligomers as well as nanoclusters (Wu et al., Science, 2010; Armando et al., 2014, FASEB J; Martinez-Munoz et al., 2018, Mol Cell) and previous data have shown that receptors with high and low affinity for CXCL12 may coexist within CXCR4 oligomers (Sohy et al., 2007, JBC). It has been hypothesized that the binding of CXCL12 to a CXCR4 protomer transmits conformational changes to the neighboring protomer, turning the latter into a low affinity receptor for the chemokine (Kufareva et al., 2014, PNAS). In this aspect, recent biophysical approaches have provided evidence that indeed CXCL12 induces conformational rearrangements within CXCR4 dimers (Perpina-Viciano et al., 2020, Mol. Pharmacol; Isbilir et al., 2020, PNAS).

Data also suggest that CXCL12 tends to increase oligomerization or nanoclustering, and that this is related to the functions of the receptor (Martinez-Munoz et al., 2018, Mol Cell; Isbilir et al., 2020, PNAS), including endocytosis (Ge et al., Sci Reports, 2017). In other receptor systems, it has also been shown that receptor dimerization is a process by which receptors that are internalized can recruit other receptors not competent for endocytosis to the endocytic machinery (Zhao, 2019, Biophys J). Although speculative, one could envisage a model where owing to oligomerization and/or nanoclustering, CXCL12-bound, high-affinity CXCR4 could drive endocytosis of other receptors that have not bound the chemokine. This could explain why endocytosis of receptors to which RES viruses bind contributes to the antiviral effects of the chemokine, even though the chemokine binds very loosely or even not at all to these receptors.

Deciphering the above hypotheses would require additional work, which, in our opinion, is well beyond the scope of the present study. In particular, the structural determinants involved in the stabilization of high and low affinity receptors should be studied, as well as the consequences on the functions and organization of the receptors. In this aspect, as noted above, it is increasingly apparent that conformational changes in CXCR4 alter the oligomeric arrangement of the receptor at the cell surface. It is therefore tempting to speculate that the different conformational constraints that stabilize CXCR4 in high- or low-affinity conformations for CXCL12 will differentially influence membrane organization of the receptor. How this will influence the capacity of CXCR4 to function as a HIV-1 coreceptor and as a therapeutic target is also unclear. Data from our laboratory and others (Vila-Coro et al., 2000, PNAS; Nakano et al., 2014, Virology; Colin et al., 2018, PLoS Pathog) suggest that CCR5 oligomerization inhibits HIV-1 entry, but whether the same is true for CXCR4 is not known. In this aspect, recent data suggest that while CXCL12 induces CXCR4 nanoclusters, which are important for CXCL12-mediated functions (e.g. chemotaxis), CD4 expression, on the contrary, tends to diminish encountering of CXCR4 molecules, thereby decreasing nanocluster formation (Martinez-Munoz et al., 2018, Mol Cell). These results are interesting in the context of our present manuscript because they suggest that under some circumstances, differences could exist in the structural requirements of CXCR4 for HIV-1 entry and CXCL12 functions.

**Page 10:**

**Binding of 4G10 to high- and low-CXCL12 affinity receptors may explain why the mAb inhibits as efficiently SENS and RES viruses (Fig. 4D).**

**But still there is ~40% of infection for both viruses in the presence of this mAb?**

The fact that mAb 4G10 inhibits only partially SENS and RES viruses is now clearly stated on page 10. We also mention that this may result from the viruses binding to receptors that are not accessible to the mAb (at least at this mAb concentration used, i.e. 10 μg/ml). Therefore, while we next show that mAb 4G10 can recognize low- and high-CXCL12 affinity receptors (**Fig 4F, G and H**), explaining why it can inhibit SENS and RES viruses (**Fig 4D and E**), these receptors likely represent only a fraction of those that are used by the viruses for entry. The notion that mAb 4G10 recognizes only a fraction of the total CXCR4 molecules on CD4TL is actually illustrated in **S5C** **Fig**, where it is shown to bind to fewer molecules of the receptor than other mAbs.

**Is there any role of CXCR7 in the antiviral assays as this receptor also (strongly) binds CXCL-12?**

**And thus there is less CXCL-12 available to bind to CXCR4?**

We have conducted new experiments to study whether CXCR7 modulates the ability of CXCL12 to inhibit HIV-1 infection of CD4 T lymphocytes. This is an interesting point raised by the reviewer, because CXCR7 has been shown to regulate CXCR4 functions through heterodimerization and by scavenging CXCL12. As a first approach to investigate this, we first measured expression of CXCR7 and CXCR4 in PHA/IL-2-activated CD4 T lymphocytes. As illustrated in **R2A Fig**, in contrast to CXCR4 that is highly expressed on CD4 T lymphocytes (see also **S6C** and **S6D Fig** in the manuscript), we detected only little to no expression of CXCR7 on these cells. We obtained similar results when we analyzed expression of both receptors in naive and memory T cell subsets, using anti-CCR7 and anti-CD45RA mAbs (data not shown). We actually found that most of CXCR7 is intracellular in CD4 T lymphocytes (**R2B Fig**), as previously shown (Hartmann et al., 2008, JLB). This suggests that most of CXCR4 does not interact with CXCR7 at the surface of CD4 T lymphocytes.

**R2A and B Fig. Expression of CXCR7 and CXCR4 in CD4 T lymphocytes.** Receptor expression levels were determined by flow cytometry using the BV421-conjugated anti-CXCR7 mAb 10D1 and the PE-conjugated anti-CXCR4 mAb 12G5, on intact cells (A), or after cell permeabilization (B).

The intracellular location of CXCR7 is actually consistent with its known property to be constitutively internalized (Naumann, 2010, PLoSOne; Luker et al., 2010, Oncogene). By so doing, CXCR7 can remove CXCL12 from the extracellular medium and thus prevents it from binding CXCR4. We thus investigated to which extent this process decreases the capacity of CXCL12 to prevent infection of CD4 T cells. To this end, we infected CD4 T cells with SENS or RES viruses in the presence or absence of CXCL12 and/or CXCL11, another chemokine which binds CXCR7 with high affinity (albeit slightly less strongly than CXCL12) and can inhibit CXCL12 from binding to the receptor (Burns et al., 2006, JEM). As shown in **R2C and D Fig**, CXCL11, even at a ten-fold higher concentration compared to CXCL12 (300 nM *vs* 30 nM), did not influence inhibition of infection. These data therefore strongly suggest than under our experimental conditions, the scavenger activity of CXCR7 is not sufficient to influence the ability of CXCL12 to inhibit HIV-1 infection of CD4 T lymphocytes.

**R2C and D Fig. Blocking CXCL12 binding to CXCR7 on CD4 T lymphocytes does not influence the antiviral capacity of the chemokine.** The infection inhibition assays by CXCL12 were carried out as in Fig 1 of the manuscript, after pre-incubation of cells with the indicated concentrations of CXCL11. Viruses pseudotyped with CXCL12-sensitive Envs (SENS, # 1, #16 and #29) or CXCL12-resistant Envs (RES, #6, #28 and #32) were used. Bars in panels C and D represent means ± SEM of two independent experiments each carried out on CD4 T lymphocytes from distinct donors. Statistics: Mann-Whitney test.

**Page 10:**

**“R5-tropic”, better to use R5 or CCR5-tropic or CCR5-using.**

We have modified the text on page 11 as requested by the reviewer.

**Page 14:**

**“… suggest that binding to CXCR4 molecules not accessible to CXCL12 is the reason why mAb 4G10 efficiently inhibits RES viruses.”**

**What epitope is 4G10 recognizing on CXCR4? Would also be useful to mention this for the other mabs used in this study.**

This information is now indicated on page 10 of the revised manuscript.
